# Evaluation of Selenomethionine Entrapped in Nanoparticles for Oral Supplementation Using In Vitro, Ex Vivo and In Vivo Models

**DOI:** 10.3390/molecules28072941

**Published:** 2023-03-25

**Authors:** Shane Forde, Giulianna Vozza, David J. Brayden, Hugh J. Byrne, Jesus M. Frías, Sinéad M. Ryan

**Affiliations:** 1 UCD School of Veterinary Medicine, UCD Conway Institute, University College Dublin, Belfield, D04 V1W8 Dublin, Ireland; 2 Environmental Science and Health Institute, Technological University Dublin, Grangegorman, D07 EWV4 Dublin, Ireland; 3 FOCAS Research Institute, Technological University Dublin, Camden Row, Dublin 8, D08 CKP1 Dublin, Ireland

**Keywords:** selenium methionine, nutraceuticals, oral delivery of micronutrients, nanoparticles, intestinal drug transport, cytotoxicity

## Abstract

Selenium methionine (SeMet) is an essential micronutrient required for normal body function and is associated with additional health benefits. However, oral administration of SeMet can be challenging due to its purported narrow therapeutic index, low oral bioavailability, and high susceptibility to oxidation. To address these issues, SeMet was entrapped in zein-coated nanoparticles made from chitosan using an ionic gelation formulation. The high stability of both the SeMet and selenomethionine nanoparticles (SeMet-NPs) was established using cultured human intestinal and liver epithelial cells, rat liver homogenates, and rat intestinal homogenates and lumen washes. Minimal cytotoxicity to Caco-2 and HepG2 cells was observed for SeMet and SeMet-NPs. Antioxidant properties of SeMet were revealed using a Reactive Oxygen Species (ROS) assay, based on the observation of a concentration-dependent reduction in the build-up of peroxides, hydroxides and hydroxyl radicals in Caco-2 cells exposed to SeMet (6.25–100 μM). The basal apparent permeability coefficient (P_app_) of SeMet across isolated rat jejunal mucosae mounted in Ussing chambers was low, but the P_app_ was increased when presented in NP. SeMet had minimal effects on the electrogenic ion secretion of rat jejunal and colonic mucosae in Ussing chambers. Intra-jejunal injections of SeMet-NPs to rats yielded increased plasma levels of SeMet after 3 h for the SeMet-NPs compared to free SeMet. Overall, there is potential to further develop SeMet-NPs for oral supplementation due to the increased intestinal permeability, versus free SeMet, and the low potential for toxicity.

## 1. Introduction

SeMet is a naturally occurring amino acid, which is an essential trace element for living organisms. The SeMet species is regarded as the principal nutritional source of selenium (Se) for higher animals and humans [1]. Since humans cannot synthesise SeMet, it meets the criteria of an essential amino acid. Although selenium in high concentrations in plasma is considered quite toxic [2], appropriate levels are considered important, since Se is involved in major metabolic pathways, antioxidant defence [3], redox reactions in detoxification and chemo-preventive pathways [1]. Moreover, Se is a micronutrient with potential pharmaceutical application in aiding treatment or the prevention of specific diseases, including cardiovascular disorders [4,5], thyroid dysfunction [6,7] and neurodegenerative diseases [8]. Subtoxic plasma levels of selenium may also have potential in cancer therapy [2,9,10,11]. Taking all these potential medical applications into consideration, the range of preventative or therapeutic dose levels of SeMet following oral administration is thought to be narrow (60–70 μg/day) [12], which makes clinical oral supplementation difficult. In addition to this, Se intake is extremely variable in populations across the world [1] owing to several factors, including the Se content of the soil in which crops are grown, Se speciation, soil pH and organic-matter content, and the presence of ions that can complex with Se [2]. Excess Se levels in plasma can also be caused by over-supplementation with Se, which can lead to severe disorders, such as selenosis in acute cases [1]. Regarding deficiency, low serum Se levels are linked to a higher risk of developing several cancer types, especially prostate, lung and colorectal cancers [10]. For instance, selenium levels below 60 μg/L have been associated with a higher risk of developing lung or laryngeal cancer [11]. Although recent studies in a variety of model systems have increased our understanding of the mechanisms of Se compounds in humans, efforts must still be made to develop formulation approaches for the oral administration of Se.

The oral administration of Se via organic forms, such as SeMet, is preferred over inorganic species due to their acceptable oral bioavailability, lower toxicity and more effective incorporation into selenoproteins in the body [13]. SeMet is organic and has a lower toxicity potential than inorganic forms, such as selenite, and this is thought to be mainly due to the differences in transformation pathways [13]. However, the oral delivery of SeMet can still be challenging due to the distinctive electronegativity and atomic radius of the Se atom (i.e., larger radius and lower electronegativity than sulphur), which makes it easier for low-valence-state Se compounds to be more readily oxidised compared to their sulphur counterparts [14]. There are few data on the intestinal absorption of SeMet in human-related models. SeMet can also undergo methylation from enzymes, such as γ-lyase, resulting in excretion and bypassing selenoprotein incorporation [15,16]. Little is also known about the effects of the brush-border enzymes and the effects of the pH environment within the epithelia of the small intestine in SeMet metabolism. It is, therefore, of interest to further investigate the intestinal stability of SeMet in the GI tract, as well as to promote more efficient permeability of SeMet across the intestinal epithelium. While SeMet is less toxic than inorganic Se, it still has a relatively low therapeutic index [17]. An oral formulation of SeMet, therefore, needs to consider the fine balance between doses that exert beneficial effects and those that may potentially be toxic.

Several formulation approaches have been investigated to improve the oral delivery of micronutrients. Prevention of stomach acid attack and enzymatic degradation through enteric coating [18] and increasing intestinal absorption via permeation enhancers [19] and nanocarrier systems [20] are potential strategies to increase the oral bioavailability of labile, poorly permeable molecules. Other methods, such as enzyme protease inhibitors (for peptides) and the use of mucoadhesive polymers, are also of interest. Often, the approach selected for the delivery system is based on the physicochemical properties of the compound. For example, if it is known that the nutrient/drug is stable in the gastrointestinal tract, then an enteric coating and protease inhibition may not be required. There are potential advantages in the use of Se-entrapping nanotechnologies to improve Se oral bioavailability by increasing the resistance to adverse pH, enzymatic cleavage and digestion [21]. The use of entrapment using chitosan and zein also gives stabilisation to Se species that may become inactivated due to the physicochemical barriers after oral administration.

Currently, there are few data available for human absorption of selenium supplements. The current study is aimed at refining knowledge of the possible obstacles related to oxidation, degradation and toxicity and how they can be overcome by encapsulation into NPs for the oral delivery of SeMet in humans. The antioxidant potential of SeMet was also investigated, while the stability of SeMet was determined in intestinal and liver homogenates. SeMet was then entrapped into nanoparticles (SeMet-NPs) consisting of chitosan, using ionotropic gelation followed by coating with zein. The cytotoxicity potential of SeMet and SeMet-NPs were measured in Caco-2 intestinal cell and HepG2 liver cell epithelia. The permeability across isolated rat small intestinal and colonic tissue mucosae was also analysed. Finally, jejunal instillation studies in Wistar rats were used to determine the capacity of SeMet and SeMet-NPs to reach rat blood plasma. The data shows that SeMet is stable in the gut and is non-cytotoxic at therapeutic concentrations. The antioxidant properties of SeMet were confirmed, and in addition, it had minimal effects on electrogenic ion secretion across isolated rat jejunal and colonic mucosae mounted in Ussing chambers. However, in vivo studies revealed that the intestinal permeability of SeMet was increased when it was entrapped in a nanoparticle (SeMet-NP).

## 2. Results

### 2.1. Antioxidant Potential of SeMet

**Figure 1:**
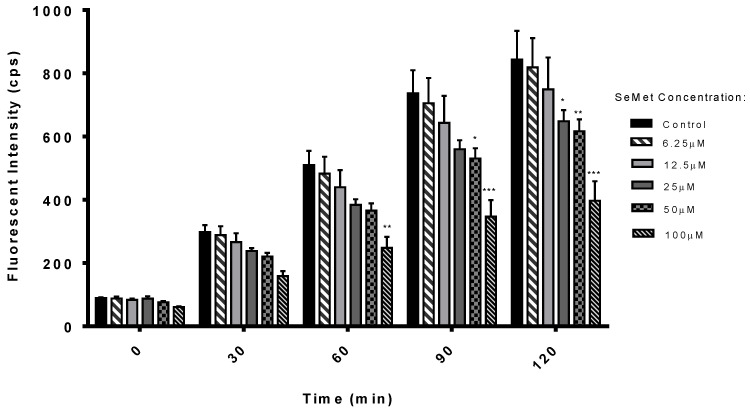
Effect of SeMet on inhibition of induction of ROS in Caco-2 cells by TBHP (100 µM). One-way ANOVA with Dunnett’s multiple comparison post-test, * *p* < 0.05, ** *p* < 0.01 and *** *p* < 0.001, compared to controls. Each value represents the mean ± SEM in triplicate (*n* = 3), with 3 independent replicates.

ROS detection in Caco-2 cells following SeMet exposure was measured as a means of testing the antioxidant properties of SeMet at concentrations between 6.25 µM and 100 µM. Cells were spiked with 100 µM tert-butyl hydrogen peroxide (TBHP) to induce oxidation in cells, followed by exposure to SeMet for 120 min. Control samples were spiked with 100 µM TBHP, with no addition of SeMet. There was a significant reduction in ROS build-up at 60 min in cells incubated with 100 µM SeMet, and further significant reductions at concentrations of 25 µM and 50 µM SeMet after 90 min and 120 min, respectively, compared to controls (Figure 1). The IC_50_ for SeMet is 13.83 ± 0.67 µM at 120 min exposure time.

### 2.2. SeMet Is Stable in Rat Intestinal and Liver Extracts

**Figure 2:**
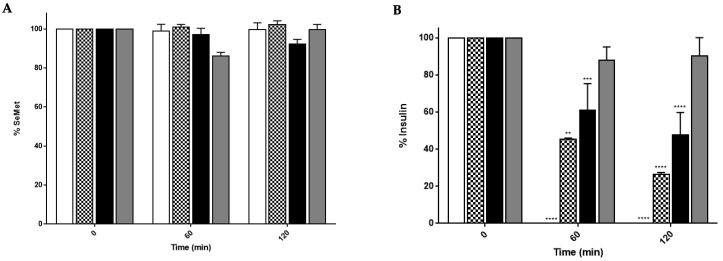
Stability of (**A**) SeMet (5 mM); (**B**) Recombinant human insulin (250 µM) in rat liver homogenates (LH—White), gut homogenates (GH—Check) and gut washes (GW—Black) and PBS control (PBS—Grey) as measured by RP-HPLC. One-way ANOVA with Dunnett’s multiple comparison; ** *p* < 0.01, *** *p* < 0.001 and **** *p* < 0.0001, respectively, compared with analyte at 0 min. Each value represents the mean ± SEM, *n* = 3.

The stability of SeMet (5 mM) in rat liver homogenates (LH), gut homogenates (GH) and gut washes (GW) was analysed by Reverse Phase High Performance Liquid Chromatography (RP-HPLC). Incubation of the SeMet in the three types of extracts showed no evidence of metabolism over 120 min, confirming the stability of the native molecule (Figure 2A). The enzymatic breakdown capacity of the three systems for peptides was confirmed by the rapid breakdown of 250 µM recombinant human insulin in each system over the same period (Figure 2B). The insulin breakdown data were similar to those of previous studies using rat gut washes [22]. The extract with the greatest capacity for metabolism by peptidases was the rat liver homogenate (LH), as indicated by the 100% breakdown of insulin at 120 min compared to approximately 70% and 50% for the GH and GW, respectively. Overall, SeMet itself is highly resistant to metabolisation by rat intestinal and liver enzymes.

### 2.3. SeMet and SeMet-NPs Are Not Cytotoxic: MTS Assay

**Figure 3:**
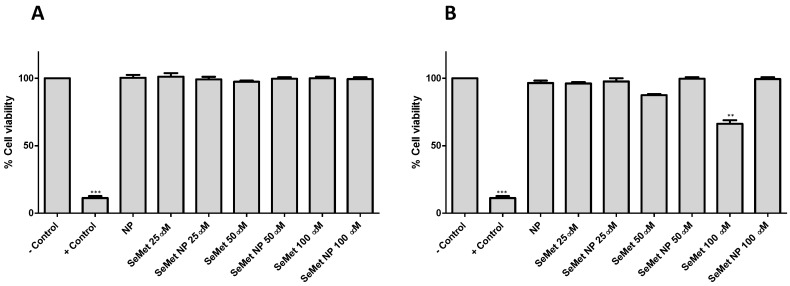
MTS assessment of SeMet, unloaded NPs and SeMet NPs exposed for (**A**) 4 h in Caco-2 cells and (**B**) 72 h in HepG2 cells. Triton^®^ X-100 (0.05%) was used as positive control and no treatment as negative control (untreated). One-Way ANOVA with Dunnett’s post-test *** *p* < 0.001, ** *p* < 0.01. Each value presented was normalised against untreated control and calculated from three separate experiments (*n* = 3), each of which included six replicates.

The formulation and characterisation of SeMet-NPs used in this study are reported by Vozza et al. [23]. In summary, SeMet-loaded Cs NPs were produced via ionotropic gelation and coated with zein. The physicochemical characteristics of SeMet-NPs are as follows: SeMet-NP diameter 377 ± 47 nm, polydispersity index 0.24 ± 0.01, zeta potential 36 ± 6 mV and encapsulation efficiency 80 ± 1.5%. Selected concentrations of SeMet and zein-coated SeMet-NPs at 25, 50 and 100 µM, along with unloaded NP controls, were assessed following acute exposure of Caco-2 cells for 4 h and chronic exposure of HepG2 cells for 72 h. Triton^®^ X-100, 0.1% (*v*/*v*), was used as the positive control. All three concentrations of SeMet in free or NP formats were non-cytotoxic following acute exposure of Caco-2 cells (Figure 3A). Similar results were found in the HepG2 cell line following chronic exposure for 72 h, with neither free SeMet nor SeMet-NPs displaying significant cytotoxicity. However, a 34% reduction in HepG2 cell viability following exposure to native SeMet at 100 µM was observed at 72 h (Figure 3B). Both loaded and unloaded NPs were, therefore, deemed to be non-toxic, according to this assay.

### 2.4. SeMet Does Not Effect Stimulated I_sc_ in Rat Jejunal and Colonic Mucosae

The effects of SeMet on the electrogenic ion transport in intestinal mucosae was assessed by measuring the change in the short-circuit current (∆I_sc_) produced by forskolin (FSK), an adenylate cyclase activator and Cl^−^ secretagogue in epithelial tissue, mounted in Ussing chambers. Rat jejunal and colonic mucosae were exposed to SeMet. Baseline I_sc_ values for untreated jejunal and colonic mucosae were 38.1 ± 7.0 and 23.3 ± 6.0 µA/cm^2^, respectively (*n* = 7, 8). Bilateral addition of 10 µM and 100 µM SeMet in both jejunum and colonic mucosae had no significant effect on FSK-stimulated Isc compared to unexposed controls (Table 1). Treatment with SeMet pre-incubated in jejunum at 10 µM and 100 µM also showed no significant decrease in I_sc_ after 30 min compared to tissues exposed to FSK only, with a similar lack of effect observed when SeMet was added at the plateau of the stimulated I_sc_ after FSK addition (Table 1 and Figures S1 and S2).

A loop diuretic, bumetanide, was also tested at the end of each experiment to confirm the nature of the ion secretion in the tissue. Bumetanide inhibits the Na-K-Cl^−^ co-transporter and is known to inhibit any changes in I_sc_ produced by FSK [24]. In total, 100 µM bumetanide was added basolaterally, 30 min after FSK addition in both the jejunum and colon, and was seen to reduce I_sc_ to the basal levels recorded prior to FSK introduction, 42 ± 8 µA/cm^2^ and 29 ± 4 µA/cm^2^, respectively (Figure S3). Overall, SeMet had no effect on stimulated electrogenic chloride sectretion transport in either region of the rat intestine, and this is further evidence that it does not impact on normal gastrointestinal physiology within the concentration range of this study.

### 2.5. Transport of SeMet across Isolated Rat Jejunal Mucosae

**Figure 4:**
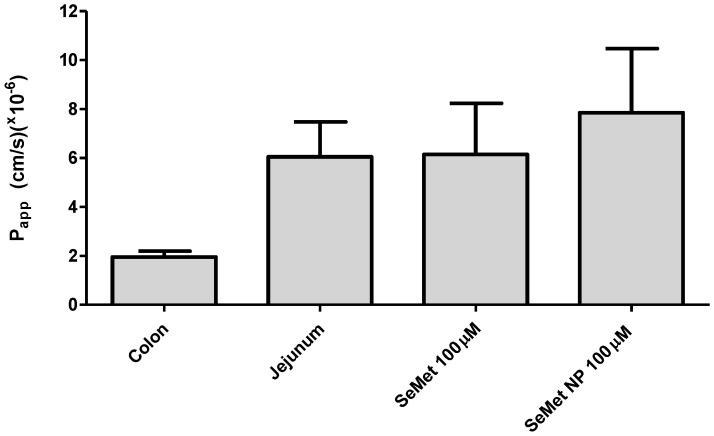
P_app_ of FD4 was measured across isolate jejunal and colonic mucosae. Effect of apical addition of 100 µM SeMet and SeMet NPs on P_app_ on jejunal mucosae with cumulative concentration of SeMet transported across the jejunum to the basolateral side over 120 min. Each value represents the mean ± EM with *n* = 3.

Transport experiments were carried out using adapted horizontal diffusion chambers derived from 1.5 mL Eppendorf^®^ tubes [25]. Initial studies of marker flux were performed to optimise and confirm that the method was effective and that the mounting of tissue was achieved without damage. FD-4 (2.5 mg/mL) was used here, as it is a common paracellular flux fluorescent marker. Figure 4 represents the FD-4 cumulative transport into the basolateral chamber and the P_app_, respectively, in both the jejunum and colon. Both tissues were untreated, and the linearity of the FD-4 flux is considered to be an important quality control feature, as its slope allows the calculation of P_app_. The basal flux of FD-4 was significantly higher in the jejunum compared to the colon, following a 2 h incubation, resulting in a total basolateral FD-4 concentration of 135 µM in jejunal-treated tissue, compared to 33 µM in colonic tissue. This is expected due to the larger surface area of the jejunum, with the majority of nutrient absorption occurring in the jejunum and ileum [26]. Similar studies in Ussing chambers have also confirmed FD-4 P_app_ to be higher in isolated rat jejunal mucosae compared to colonic mucosae [27].

SeMet or SeMet-NPs were added apically to the modified chambers at 100 µM concentrations of SeMet, samples being taken basolaterally at 20 min intervals over a 2 h period. Figure 4 shows the cumulative concentration of the SeMet detected in the basolateral chamber. SeMet (100 µM, donor side) in both free and NP formulations was transported across isolated rat jejunum in similar quantities, both formats resulting in 20–25 µM SeMet in the basolateral chamber at 2 h. SeMet (100 µM) produced an average P_app_ of 5.36 × 10^−6^ cm/s compared to 8.55 × 10^−6^ cm/s for SeMet-NPs, indicating an increased trend for the permeability of SeMet across the jejunum from the latter. Similar SeMet transport studies were carried out on isolated rat colonic mucosae; however, SeMet was not detectable on the basolateral side.

**Figure 5:**
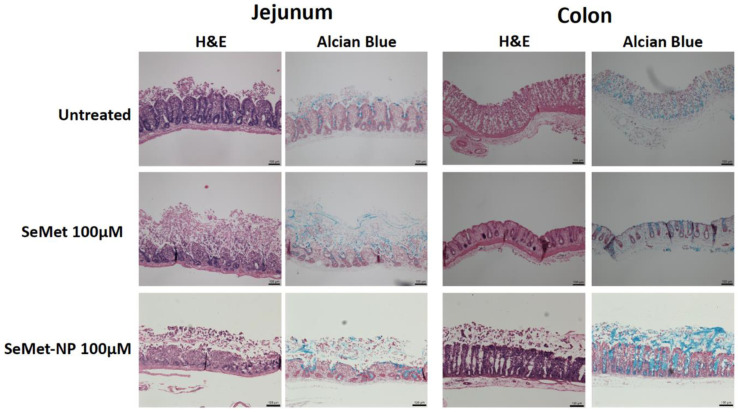
Representative H&E and Alcian Blue staining of rat jejunal and colonic mucosae in adapted horizontal diffusion chambers following a 2 h exposure with SeMet and SeMet-NPs compared to untreated tissue. Black arrows indicate erosion of epithelial layer. Horizontal bars in each panel = 100 µm.

Histological samples of treated and untreated rat jejunal and colonic tissues were assessed following the transport studies in adapted horizontal diffusion chambers at 2 h. Jejunal sections, including untreated controls, displayed varying levels of epithelial surface sloughing, which may be a result of mechanical damage during tissue processing as opposed to a treatment effect. The exposure of jejunum to 100 µM of SeMet showed little damage, while SeMet-NPs induced moderate erosion of epithelia, as indicated by black arrows (Figure 5). Colonic mucosae samples, on the other hand, had a more intact tissue profile than jejunal tissue, per se, all samples showing little damage following 2 h incubation. Slight sloughing can be seen in the treatments of SeMet and SeMet-NPs, although, compared to the control, there does not seem to be significant damage caused to the tissue sections (Figure 5). The damage seen in jejunal sections is not uncommon in ex vivo studies, as the tissue is more delicate than colonic mucosae and undergoes smooth muscle stripping prior to mounting, which increases the mechanical impact on the tissue. The results from both tissues indicate neither treatment concentration is cytotoxic to either rat-isolated jejunal or colonic tissue, which is consistent with the in vitro cytotoxicity results for Caco-2 cells.

### 2.6. In Vivo Studies

**Figure 6:**
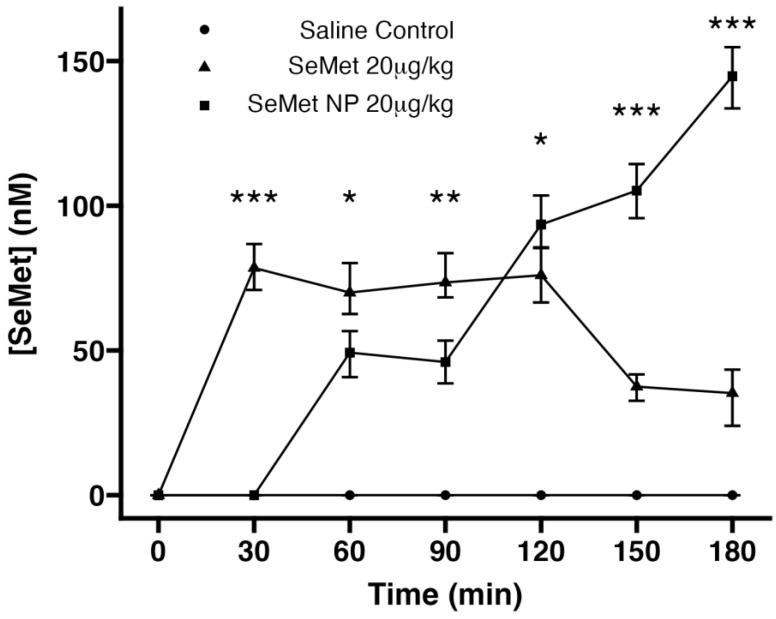
Measurement of plasma SeMet concentration following jejunal instillation of SeMet, SeMet-NPs and saline. Bonferroni test *** *p* < 0.001, ** *p* < 0.01, * *p* < 0.05, SeMet NPs compared to SeMet. Each value represents the mean ± SEM, *n* = 5 independent experiments.

Native SeMet reached a peak plasma concentration after 30 min (82 nM) in rat intestinal instillations following a dose of 20 µg/kg. Initial detection of SeMet from the instilled NP formulation was from 30 min onwards, suggesting a delay compared to the free SeMet. There was a linear trend, indicating an increase to a higher concentration than native SeMet treatment after 180 min (to 145 nM) compared to native SeMet treatment (41 nM) (Figure 6), with no sign of a pleateau effect for SeMet-NPs. No SeMet was detected in the saline-treated group.

**Figure 7:**
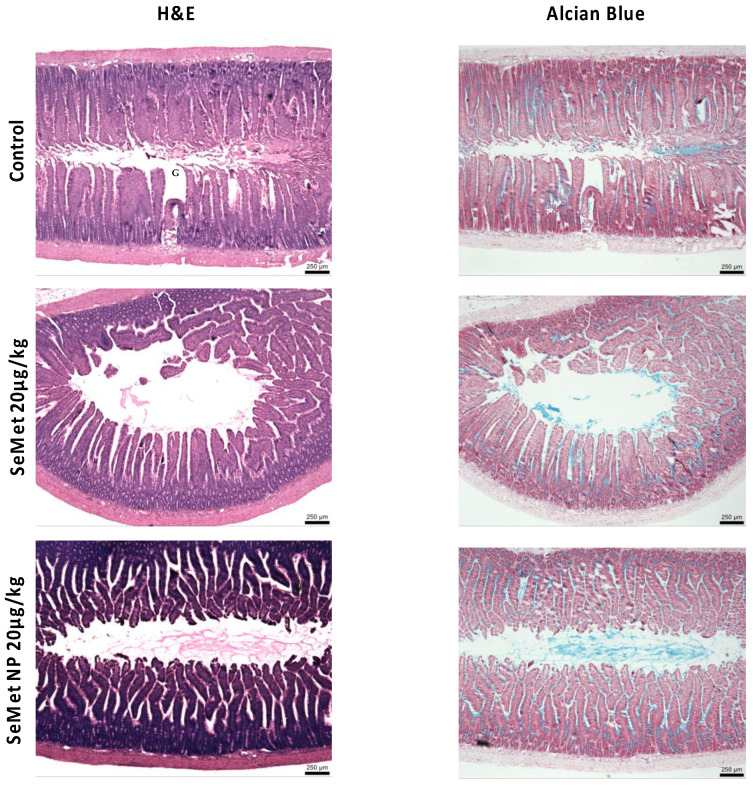
H&E and Alcian Blue and neutral red staining of mucosae 180 min after intra-jejunal instillations. Groups: PBS control, free SeMet (20 µg/kg), SeMet NP (20 µg/kg). Images captured using a light microscope. Goblet cells are indicated by ‘G’. Black arrows show epithelial sloughing. Horizontal bars on panels = 250 µm.

The effects of SeMet and SeMet-NPs on jejunal tissue following jejunal instillations were examined by histological analysis following 180 min exposure (Figure 7). Control jejunal tissue showed an intact epithelium with healthy goblet cells. Exposure to SeMet (20 µg/kg) induced no damage to the epithelium or jejunal villi. SeMet-NPs (20 µg/kg of SeMet) induced some cellular sloughing of jejunal villi, although the overall physiological structure was retained. Overall, the histology is consistent with the cytotoxicity data for Caco-2 cells and the rat jejunal tissue analysis from adapted horizontal diffusion chambers, indicating the absence of intestinal tissue damage in the presence of either SeMet or SeMet-NPs.

## 3. Discussion

A better understanding of the potential obstacles and mechanisms of intestinal absorption for oral supplementation of SeMet is required. The focus of this work was the assessment of the cytotoxicity and stability of SeMet, with a specific focus on the uptake of SeMet within the body using in vitro, ex vivo and in vivo models of assessment. The antioxidant potential was first investigated. An inverse relationship between the SeMet concentration and fluorescent intensity was observed in the ROS assay, suggesting a concentration-dependent reduction in the build-up of peroxides, hydroxides and hydroxyl radicals via scavenging. The antioxidant potential of SeMet has also been demonstrated using other assays, such as 1,1-diphenyl-2-picryl-hydrazyl, cupric-reducing antioxidant capacity and the Folin–Ciocalteu assay [28]. Miranda et al. indicated SeMet treatment as low as 10 nM on isolated epithelial cells can modulate apoptosis and proliferation, independent of a selenoprotein-mediated reduction of hydrogen peroxide [29]. Zhang et al. also observed that SeMet activates the Nrf2/HO-1 signalling pathway, which increased the expression of downstream antioxidant enzymes and reduced the content of ROS in tissues [30]. Recently, Ren et al. demonstrated that SeMet can enhance the antioxidant capacity of pig kidney epithelial (LLC-PK) cells after porcine deltacoronavirus infection [30]. LLC-PK cells were infected with porcine deltacoronavirus, which reduced the activity of plasma glutathione peroxidase (GSH-PX) significantly (0.01 < *p* < 0.05) [31]. SeMet-NP samples were not included, as the incubation time for test samples on Caco-2 is 1 h for the ROS assay. In contrast, the controlled release of SeMet from SeMet-NPs is over the timescale of 2–6 h. The in vitro cell-based ROS assay is, therefore, not suitable for measuring the antioxidant effect of SeMet-NPs with a controlled release mechanism. However, the results of the ROS assay provide further evidence in support of the potential of SeMet as an antioxidant.

Organic Se species have been widely reported to be less toxic than inorganic forms [32,33]. However, little has been published on the specific cytotoxicity potential of SeMet species in human intestinal and hepatic epithelial cell types. This is of particular interest when considering a potential oral formulation for SeMet. Neither SeMet nor SeMet-NPs induced cytotoxicity in Caco-2 cells following short-term exposure (4 h) over the selected concentration range (25–100 µM). For HepG2 exposures over 72 h, no cytotoxicity was observed for SeMet-NPs at all concentrations tested. Low concentrations (25 and 50 µM) of native SeMet showed no cytotoxicity either, although a reduction in HepG2 cell viability at 100 μM SeMet was observed (approx. 66% cell viability). Time points were selected with the intention of mimicking in vivo conditions for each cell type [34,35]. Similar results were observed by Takahashi et al. [17], whereby SeMet elicited no significant change in the viability of Caco-2 cells, although it did show marginal toxicity to HepG2 cells at concentrations > 80 μg/mL after prolonged exposure. Kajander et al. also showed SeMet toxicity at concentrations ≥ 40 μM in various hepatoma cell lines [36]. SeMet-NPs elicited no significant reduction in the viability of either cell line at equivalent concentrations (100 μM), indicating that, by encapsulating SeMet within the chitosan NP matrix, potential cytotoxic effects of high concentrations of SeMet could be reduced. The nanoparticle design can, therefore, provide protection from cytotoxicity if high concentrations of SeMet are required for oral administration. It has previously been shown that chitosan-based nanoparticles can reduce the toxicity of inorganic Se [37].

Due to conflicting literature on the metabolism pathways for SeMet, we assessed the integrity of SeMet using ex vivo intestinal and liver models. SeMet remains intact following incubation in excised rat liver and intestinal homogenates and intestinal luminal fluid. Confirmation of the peptidase activity of the three bioassays was apparent from the detected breakdown of insulin. Simulated intestinal fluids are used as a more physiological in vitro system incorporating modified pH and surfactants, although they still underestimate the potential breakdown [38], as they do not include brush-border and intracellular peptidases. The intestinal GW and GH used incorporate these enzymes and may provide an alternative prediction of the stability of these peptides against gastrointestinal peptidases in vivo. The ex vivo gastrointestinal fluids include secretory digestive enzymes in the GW and brush-border peptidases, intracellular enzymes and appropriate pH in GH [39]. GW displayed a higher degradation of insulin than in LH or GH, which was comparable to similar studies [22,40]. As the GW was produced by flushing the small intestinal segment with simulated intestinal fluid san pancreatin (SIFsp), the resulting fluid is buffered to a pH favourable for digestive enzymes, whereas the tissues were homogenised in an unbuffered, physiologically relevant salt solution. Therefore, GW and GH can be used as complementary methods of determining the oral stability to intestinal pH and secretory and/or intracellular peptidase enzymes. It was further concluded that SeMet is resistant to metabolism by liver enzymes and intestinal pH, suggesting that SeMet is not degraded during first pass metabolism and can be transported into the systemic circulation intact. Due to SeMet showing stability in the intestine and liver, SeMet-NPs were not included in these assays.

It has previously been reported that selenium deficiency in the diet is associated with chronic diarrhoea in male adults [41] and with protracted diarrhoea in children [42,43]. Our study used the same ex vivo intestinal Ussing chamber model as Bzik et al. [44], who studied the mechanism of the antidiarrheal action of zinc on rat intestinal epithelia. The cAMP-regulated electrogenic chloride secretion in intestinal epithelia can be mimicked ex vivo by using FSK to activate adenylate cyclase, using measurement of TEER and I_sc_ as indirect assays of the activity in isolated mucosae. The model permits investigation of any anti-secretory effects of SeMet, as well as calculation of apical-to-basolateral SeMet flux. The I_sc_ is defined as the current flow through the tissue when the voltage across the tissue is zero or ‘short-circuited’ [45]. Magnitudes of change in I_sc_ were determined before and after basolateral addition of FSK, in both isolated jejunal and colonic mucosae. Basal I_sc_ values for the rat jejunum (38 ± 7 µA/cm^2^) and colon (23 ± 67 µA/cm^2^) were similar in range to previously reported values [44,46]. Addition of SeMet to both the apical and basolateral sides of jejunal mucosae prior to FSK introduction resulted in no significant differences in the simulated I_sc_ increase compared to the control. While there was a tendency for the ΔI_sc_ to be less than that of the FSK control treatment, when considering the high concentrations used in this study, it does not suggest a loss in the secretory capacity for intestinal tissue. A similar trend was seen when SeMet was added post-FSK, resulting in a non-significant ΔI_sc_ compared to FSK alone. Colonic mucosal studies show a more striking increase in I_sc_ following FSK treatment compared to jejunum, with FSK-only controls displaying a 79% and 225% increase for jejunum and colonic tissue, respectively, with peak I_sc_ levels reaching 120 µA/cm^2^. This may be due to the low residual Cl^−^ present in the colon [47]. This ΔI_sc_ is also consistent with the studies of Bzik et al., who reported FSK stimulated increases of up to 110 µA/cm^2^ for FSK-only controls [44]. SeMet at 10 µM and 100 µM did not have a significant effect after 30 min following FSK treatment in isolated rat colon compared to controls. Similar data were seen when SeMet was introduced after FSK addition, suggesting negligible anti-secretory changes induced by SeMet in either tissue.

To confirm that the FSK-induced increase in I_sc_ was due to Cl^−^ secretion, bumetanide was added basolaterally, acting as an inhibitor of Cl^−^ secretion from epithelial tissue via blockage of the Na^+^-K^+^-Cl^−^ co-transporter. An immediate reduction of I_sc_ back to basal values upon bumetanide addition in both jejunal and colonic mucosae is seen (Figure S3). These results are consistent with previous studies investigating the effects of Cl^−^ secretion on isolated rat intestinal tissue using the Ussing chamber model [47].

The horizontal adapted diffusion chamber model was used to assess the intestinal transport of SeMet and SeMet-NPs [25,48]. The classical Ussing chamber was not used due to the orientation of mounted tissue in the chamber, resulting in continuous circulation of nanoparticles in a diluted reservoir within the system, along with the glycocalyx restricting the direct contact of nanoparticles with the cells of the villus epithelium [49]. FD-4 transport across isolated jejunal and colonic mucosae was achieved, the P_app_ values being consistent with those of previous studies using Ussing chambers [50]. The P_app_ for SeMet across rat intestinal tissue was similar to that reported by Gammelgaard et al. in Caco-2 monolayers [51]. The transport pathway for SeMet is thought to be transcellular, and it uses the same apical transporters as sulphur analogues. In addition, SeMet can be stored in intracellular proteins, which represents an Se supply in case of shortage [12]. The P_app_ of SeMet, when presented as SeMet-NPs, was 59.5% higher compared to free SeMet, indicating enhanced delivery across the epithelium due to nanoparticle entrapment. High concentrations of SeMet and SeMet-NPs on the apical or basolateral side of rat intestinal tissue did not affect the histology of the tissue, confirming no induction of damage.

In vivo models are essential to confirm the delivery and uptake of SeMet. Jejunal instillations were used as an initial screen to allow test agents to get close to the gut wall in a high concentration without dilution. The bioavailability in rats was determined for SeMet in both native and NP form at a dose of 20 µg/kg, administered via injection to rat jejunal loops. After administration, plasma concentrations peaked after 30 min and remained at a similar concentration between sampling intervals up to 120 min. The peak plasma concentration was seen at 120 min, similar to that of Zhang et al. [52], after which levels began to decrease due to SeMet distribution and metabolism, or from incorporation into proteins, or storage in the methionine pool, along with a small amount of excess SeMet excreted in the urine [53]. However, when encapsulated in the coated NP, a delayed appearance in plasma was observed, followed by a 3.5-fold higher plasma SeMet concentration after 180 min compared to free SeMet, and with no sign of the SeMet concentration plateauing. Overall, the significant improvement in delivery of SeMet from the NP could be explained by the protection of the nanoparticle provided by zein, along with the sustained release close to the epithelium. Endogenous SeMet was not detected in rat plasma prior to administration and was also not detected at any time point in the saline group, which is consistent with previous studies in both rats and humans [54,55]. Jejunal instillations give the best chance of absorption and are a benchmark for further in vivo work with SeMet-NPs. Future studies will progress to oral gavage, which will bring into play additional variables of the gastric breakdown of the SeMet-NPs, dilution and gastrointestinal transit. It will also be considered to use a pH-sensitive polymer-coated capsule as the final dosage form if the zein does not sufficiently control the release. This would allow the NPs to be released directly in the upper small intestine.

Histological samples from in vivo studies show intact tissue in all three test groups, with no damage seen in jejunal sections excised after the final plasma samples were taken. This is consistent with the in vitro cytotoxicity and the ex vivo tissue studies, confirming the innocuous nature of the NP. Furthermore, the dose of SeMet used in the in vivo study (20 µg/kg) was the same as that used by Zhang et al. [52] and represents a supra-nutritional concentration. Overall, the improvement, in terms of the delivery to plasma, of SeMet from NPs may be explained by the protection and sustained release provided by zein and chitosan.

## 4. Materials and Methods

### 4.1. Reagents and Chemicals

The chitosan ultrapure PROTASAN™ UP (CL113, Mw = 110–150 kDa, DDA = 85%, Endotoxins ≤ 100 EU/g, Heavy metals ≤ 40 ppm) was purchased from NovaMatrix (Sandvika, Norway). DL-selenomethionine, D(+)-Trehalose dihydrate and zein, of ≥99% purity, were obtained from ACROS Organics™, Fisher Scientific, (Dublin, Ireland). Ultra-pure water 18 mΩ·cm^−1^ was obtained from a Millipore simplicity 185 model instrument, (Watford, UK) and was used for all aqueous solution preparations throughout. Sodium Tripolyphosphate (TPP) of technical grade (85%) and all other reagents, chemicals and solvents were analytical grade from Sigma-Aldrich (Wicklow, Ireland). Caco-2 and HepG2 cells were obtained from European Collection of Cell Cultures (Salisbury, UK).

### 4.2. ROS Assay

Caco-2 cells were seeded on a 96-well plate at a density of 2.5 × 10^4^ cells/well in DMEM (phenol red free) and incubated at 37 °C for 24 h. Cells were then treated with SeMet (6.25–100 µM) and dissolved in DMEM (phenol red free) at 200 µL/well for 1 h. Cells were then washed, and dichlorodihydrofluorescein diacetate (DCF-DA) was added for 45 min at 37 °C in the dark. DCF-DA was removed and replaced with 1× buffer, with the cellular ROS accumulation quantified using a fluorescence plate reader Spectramax Gemini, Molecular Devices (San Jose, CA, USA). Each plate was measured at an excitation of 485 nm and emission 525 nm. The buffer was removed and replaced with a positive control, TBHP, for 2 h [56], with the absorbance measured at intervals of 0, 30, 60, 90 and 120 min.

### 4.3. Preparation of Ex Vivo Rat Gastrointestinal Enzyme Fluids and Liver Homogenates

Studies were carried out in accordance with the UCD Animal Research Ethics Committee protocol (AREC 14–28-Brayden) and in adherence with the “Principles of Laboratory Animal Care” (NIH Publication #85-23, revised in 1985). Male Wistar rats (250–300 g) Charles River (Canterbury, UK) were euthanised by stunning and cervical dislocation under the UCD Animal Research Ethics Committee (Protocol: AREC 14–28 Brayden). A section of the jejunal region of the small intestine was excised (~15 cm) and flushed with simulated intestinal fluid san pancreatin (SIFsp). SIFsp was prepared as per United States Pharmacopoeia: a 25 mM KH_2_PO_4_ buffer solution at pH 6.8 [57]. The fluid was flushed through the intestine, collected and extracted three to four times with 5 mL ice-cold dichloromethane to remove lipids. The final extract was filtered using Millipore™ 0.45 µm syringe filters [58], providing a fluid designated as ‘gut wash’ (GW). The initial tissue was then sliced into ~0.5 g pieces and inserted into 2 mL microtubes, before being homogenised in HBSS at 30 Hz for 2 min using a Qiagen TissueLyser^®^. The homogenate was centrifuged at 10,000 rpm for 5 min, and the supernatant was filtered using 0.45 µm. Aliquots of the filtrate were centrifuged further in 0.22 µM cellulose acetate centrifuge tube filters, before a final syringe filtration was carried out. This final filtrate was designated ‘gastrointestinal homogenate’ (GH). A fresh liver (~7–10 g) was harvested from an euthanised Wistar rat, placed in ice-cold saline and homogenised in 10 mM PBS pH 7.4 at 30 Hz for 2 min, as per GH. The homogenate was centrifuged 10,000 rpm at 4 °C for 5 min, and the supernatant collected was designated as ‘liver homogenate’ (LH). Each gut wash and homogenate were prepared using three independent replicates.

### 4.4. Stability Studies in Isolated Rat Intestinal and Liver Extracts

SeMet (4 mM) was incubated in LH, GH and GW for 120 min at 37 °C and agitated on a shaker at 150 rpm. Recombinant human insulin (250 µM) was incubated in these buffers as a positive control to confirm that active degradative enzymes were present in each extract [58]. Samples of 100 µL were taken at T = 0, 15, 30, 60, 90 and 120 min for SeMet samples, and at T = 0, 60 and 120 min for insulin samples, before immediately centrifuging at 10,000 rpm at 4 °C, transferring to glass HPLC vials, Apex Scientific (Dublin, Ireland) and placing on dry ice to stop the reaction. Note that the vials contained inserts to ensure the appropriate volume level was reached for instrument detection. All samples were analysed via RP-HPLC, with each having 3 replicates each from 3 different experiments.

### 4.5. Reverse-Phase HPLC Analysis

Standard dilutions of SeMet were prepared in HBSS buffer, while insulin standards between 62.5 and 500 µM were prepared by dissolving in 0.1 M HCL, followed by HBSS with pH-adjusted back using 0.1 M NaOH. SeMet samples were analysed on an Agilent 1200 series HPLC, using a Luna 5 µ C18 column 25 cm × 4.6 mm Hypersil GOLD, Thermo Scientific (Horsham, UK). Samples were measured using an isocratic elution of mobile phase (2% MeOH, 0.1% TFA, 97.9% dH_2_O water), at a flow rate of 1 mL/min. The sample run time was 20 min, with an injection volume of 20 µL and UV absorbance 218 nm. The tray temperature was maintained at 45 °C. The mobile phase for detection of recombinant human insulin contained 0.1% TFA, 30% Acetonitrile and 69% dH_2_O water. The flow rate was 1 mL/min at 15 min per sample. The injection volume was 5 µL and the UV absorbance 215 nm. Note that samples were kept on ice for a maximum of 10 min before analysing by HPLC to ensure there was minimal degradation. The results were analysed using HPLC software ChemStation Windows.

### 4.6. Synthesis of SeMet-NPs

SeMet-entrapped NPs were produced using a modified ionic gelation method [59]. Briefly, Cs was dissolved in buffered pH medium (3, 4 or 5 pH) at a concentration of 3 mg/mL and filtered through a 0.22 μm syringe filter Millex Millipore (Poole, UK) to remove undissolved Cs. A known amount of SeMet was then added to the Cs solution prior to crosslinking to obtain a final load concentration of 0.05, 0.150 or 0.250 mg/mL. TPP was added dropwise to the solution under stirring at 700 rpm and room temperature to yield final mass ratios of Cs:TPP NPs of 4:1, 6:1 or 8:1. All of these experimental parameters (pH, concentrations and ratios) were prepared according to the Box–Behnken Design [23]. The NP suspension was stirred at 700 rpm for 30 min at room temperature for further crosslinking. After stabilisation, NPs were then transferred to a 30 kDa molecular-weight cut-off centrifugal filter and isolated by centrifugation at 3000 rpm for 30 min. Filtered H_2_O (equivalent in volume to the recovered supernatant) was then added to the isolated NPs and sonicated at a 35% amplitude for 30 s with 5 s pulse intervals. Physicochemical properties of the NPs were then determined, using a Malvern Zetasizer NanoZS (Worcestershire, UK), and the supernatant was retained for EE% determination, as outlined by Vozza et al. [23]. The optimised formulation has a mass ratio of 6:1 (Cs:TPP), Cs media (pH 5) and a final SeMet load concentration of 0.15 mg/mL. The Box–Behnken experimental design of experiments (DoE) was applied for the formulation and optimisation of the key variables associated with SeMet-NPs coated with zein. The physiochemical characterisation and controlled release studies were reported previously by Vozza et al. [23].

### 4.7. MTS Assay

Caco-2 and HepG2 cells at a density of 2 × 10^4^ cells/well were cultured on 96-well plates in DMEM and EMEM, respectively; supplemented with 10% foetal bovine serum, 1% L-glutamine, 1% penicillin-streptomycin and 1% non-essential amino acids; and incubated at 37 °C in a humidified incubator with 5% CO_2_ and 95% O_2_. The assay was carried out using a 4 h exposure time for the SeMet test samples on Caco-2 cells, and 72 h on HepG2 cells, using Triton™ X-100 (0.05%) as a positive control. Cells were treated with MTS (3-(4,5-dimethylthiazol-2-yl)-5-(3-carboxymethoxyphenyl)-2-(4-sulfophenyl)-2H-tetrazolium accordingly. Optical density (OD) was measured at 490 nm. Each value presented was normalised against the untreated control and calculated from three separate experiments, each of which included six replicates.

### 4.8. Electrophysiology of Rat Intestinal Tissue Mucosae

Studies were carried out in accordance with the UCD Animal Research Ethics Committee protocol (AREC 14–28-Brayden) and in adherence with the “Principles of Laboratory Animal Care” (NIH Publication #85-23, revised in 1985). Male Wistar rats (250–300 g; Charles River (Canterbury, UK) were euthanized by stunning and cervical dislocation. Colonic segments were removed and placed in freshly oxygenated Krebs–Henseleit buffer (KH) at pH 7.4 at 37 °C. Excised intestinal tissue was dissected and muscle-stripped, according to previous methods [60]. Mucosae were mounted in Ussing chambers with a circular window at 0.63 cm^2^ [61]. Then, 5 mL KH was added bilaterally. KH was oxygenated using a gas-lift system with 95% O_2_/5% CO_2_. Each chamber half had a voltage (V) and current (I) Ag/AgCl electrode, which were connected to a pre-amplifier (Pre-Amp), and all four Pre-Amps were connected to the voltage clamp apparatus EVC4000; WPI (Stevenage, UK). The potential difference (PD, mV) was measured across the mucosa in an open-circuit configuration. When the voltage was clamped to 0 mV, the short-circuit current (Isc, mA/cm^2^) was determined. After 20 min equilibration, the tissue transepithelial electrical resistance (TEER, Ω·cm^2^) was determined using Ohm’s Law (30 Ω·cm^2^ and 70 Ω·cm^2^ for the rat jejunum and colon, respectively). Tissues were pre-incubated with SeMet (10 µM and 100 µM), added both apically (AP) and basolaterally (BL), before the addition of forskolin (FSK). In other experiments, FSK was added first and BL with the addition of SeMet (AP + BL) on the plateau of the FSK-induced peak. After the additions, the ∆I_sc_ was recorded as an indirect measure of the electrogenic anion secretion across the mucosae.

### 4.9. Isolated Rat Intestinal Mucosae Transport Studies Using Adapted Horizontal Diffusion Chambers

This system is adapted from the classic Ussing chamber (method 4.8) and described by Soni et al. [25]. The tissue (jejunum or colon) was prepared, muscle-stripped and mounted onto the horizontal diffusion chamber. The cap was snapped into the tube, creating a tissue barrier. The mounted tissue was lowered into the Sterilin™ tube within a water bath and heated to 37 °C for 15 min. The basolateral compartment was gassed with 95% O_2_/5% CO_2_. At time zero, SeMet, SeMet-NPs and FITC-dextran 4000 Da (FD_4_) were added to the apical chamber. Basolateral samples were taken every 20 min for 120 min, and apical samples were taken at 0 and 120 min in order to calculate the apparent permeability coefficient (P_app_). Withdrawn samples were replaced with an equal volume of fresh KH. FD_4_ samples were added to a 96-well microplate with a signal intensity of FD_4_, measured using a Spectrofluorometer MD Spectramax Gemini with an excitation wavelength of 490 nm and emission wavelength of 525 nm. SeMet and SeMet-NP samples were measured by LC-MS. The P_app_ (cm·s^−1^) was calculated according to the following equation:

(1) $$P_{\mathrm{app}}=\frac{\mathrm{dQ}}{\mathrm{dt}}\frac{1}{A\cdot C_{0}}$$

where dQ/dt is the transport rate across the epithelium (slope of the cumulative amount of SeMet versus time) (mol/s); A is the surface area (0.63 cm^2^); C_0_ is the initial concentration of SeMet in the apical compartment (mol/mL). Intestinal mucosae transport experiments were carried out as four independent replicates.

### 4.10. LC-MS Analysis

The quantification of SeMet was adapted from a method by Zhang et al., 2018 [52]. The analysis was performed on an Agilent-Poroshell 120 EC-C18, 2.1 mm × 150 mm, 4 µm particle size UPLC column, using a mobile phase consisting of water:acetonitrile:formic acid (99:1:0.1, *v*/*v*/*v*) at a flow rate of 0.3 mL/min for a total run time of 8 min. The autosampler was conditioned at a set temperature of 20 °C, and the injection volume was 5 µL. The UPLC system was coupled to an Agilent 6400 series Triple Quadrupole LC/MS system (LC-QQQ). Quantification was performed using the multiple reaction monitoring (MRM) mode to monitor the precursor product ion transitions of *m/z* 198.0 > 181.1 for SeMet. The capillary voltage was set at 3.5 kV, with a source temperature of 120 °C and a desolvation temperature of 350 °C. Nitrogen gas flows were set at 700 L/h and 50 L/h for the desolvation and cone gases, respectively. Argon was used as a collision gas and was introduced at 0.15 mL/min. The cone voltage for SeMet was 14 V and the collision values set at 12 eV.

### 4.11. Histology

For experiments with jejunal tissue and colonic mucosae exposed to SeMet test samples, tissues were removed after 120 min exposure in Ussing chambers and Adapted Horizontal Diffusion Chambers and immersed in 10% (*v*/*v*) buffered formalin for 48 h. Tissues were stained with haematoxylin and eosin (H&E) and Alcian blue. Slides were visualized under a light microscope, NanoZoomer 2.0-HT light microscopy and images were taken with a high-resolution camera, Micropublisher 3.3 RTV, QImaging and Image-Pro^®^ Plus version 6.3 acquisition software.

### 4.12. In Vivo Instillation Studies in Rats

All procedures involving animals were carried out under the guidelines outlined by the National Centre for the Replacement, Refinement & Reduction of Animals in Research (NC3R) and adhered to by the revised EU Directive 86/609/EEC for the use of animals in experiments and other scientific purposes. Rat jejunal instillations were carried out in compliance with the Health Products Regulatory Authority (HPRA) project license number AE18982/P140. Male and female rats weighing between 300–350 g were used in the in vivo experiments in a 50:50 ratio for each treatment to ensure no gender bias in the study. Animals were housed in a controlled environment, with a set temperature and humidity and a 12:12 h light/dark cycle. The rats had access to standard laboratory chow and water ad lib and were fasted 20 h before undergoing jejunal instillations, with free access to water.

### 4.13. Anaesthesia and Euthanasia

Anaesthesia was induced with isoflurane (Iso-Vet, 1000 mg/g isoflurane liquid for inhalation, Piramal Healthcare (Morpeth, UK), at a flow rate of 5 L/min and oxygen flow at 4 L/min, in an induction chamber. The rat weights were recorded before the anaesthetic was switched to face masks, Blease Medical Equipment Ltd. (London, UK), and maintenance of the anaesthetic involved conditions of reduced isoflurane flow to 2.5 L/min and oxygen flow at 2 L/min, with animals kept on a heating pad set at 37 °C. Animals were euthanised after the final blood sample was taken by the intra-cardiac injection of 0.3–0.4 mL pentobarbital sodium, Euthatal™, Merial Animal Health Ltd. (Essex, UK).

### 4.14. Jejunal Instillations

Jejunal instillations were carried out by modifications described by Aguirre et al., 2014) [62]. Under anaesthetic, a midline laparotomy was performed, and the mid jejunum was identified. A section of jejunum of ~6 cm was tied off at both ends before a solution of SeMet/SeMet-NPs (30 µg SeMet/mL) dissolved in Dulbecco’s Phosphate Buffered Saline (DPBS) was injected into the lumen with a 30 G needle. The volumes of solutions injected varied between 200 and 250 µL, depending on the weight of the animal, with dosages set at 8 µg SeMet/kg body weight. Blood samples (~300 µL) were taken via retro-orbital bleeds at T = 0, 15, 30, 60, 90, 120, 150 and 180 min, collected in 1 mL heparinised tubes and stored on ice before centrifuging at 6500× *g* for 5 min. Blood plasma was transferred to Eppendorf tubes and analysed for SeMet by LC-QQQ. Following the euthanasia of the animal, the targeted jejunal section was excised and immersed in 10% (*v*/*v*) formalin for 48 h and processed. Tissues were stained with haematoxylin and eosin (H&E) and Alcian blue. Slides were visualized under a light microscope NanoZoomer 2.0-HT light microscopy and images were taken with a high-resolution camera Micropublisher 3.3 RTV, QImaging and Image-Pro^®^ Plus version 6.3 acquisition software.

## Figures and Tables

**Table 1:** Effect of SeMet on FSK-stimulated ∆I_sc_ in rat jejunum and colonic mucosae.

Treatment	ΔI_sc_ Jejunum	ΔI_sc_ Colon
FSK only (10 µM)	79	225
Preincubation with SeMet (10 µM) + FSK	64	164
Preincubation with SeMet (100 µM) + FSK	38	168
FSK + SeMet (10 µM) at plateau	44	183
FSK (BL) + SeMet (100 µM) at plateau	15	134

## Data Availability

Not applicable.

## Supplementary Materials

The following supporting information can be downloaded at: https://www.mdpi.com/article/10.3390/molecules28072941/s1, Figure S1: The effect of SeMet on FSK-mediated Cl^−^ secretion in rat jejunum mucosae.; Figure S2: The effect of SeMet on FSK-mediated Cl^−^ secretion in rat colonic mucosae and Figure S3: Bumetanide response to FSK stimulated Cl^−^ secretion.
