# Supplementary material for: Evaluation of Selenomethionine Entrapped in Nanoparticles for Oral Supplementation Using In Vitro, Ex Vivo and In Vivo Models
Source: Molecules. 2023 Mar 25;28(7):2941. doi: 10.3390/molecules28072941 (PMC10095941; doi:10.3390/molecules28072941)
Supplement: Supplementary file 1 [file molecules-28-02941-s001.zip › molecules-2286198-supplementary.pdf]

Supplementary

A

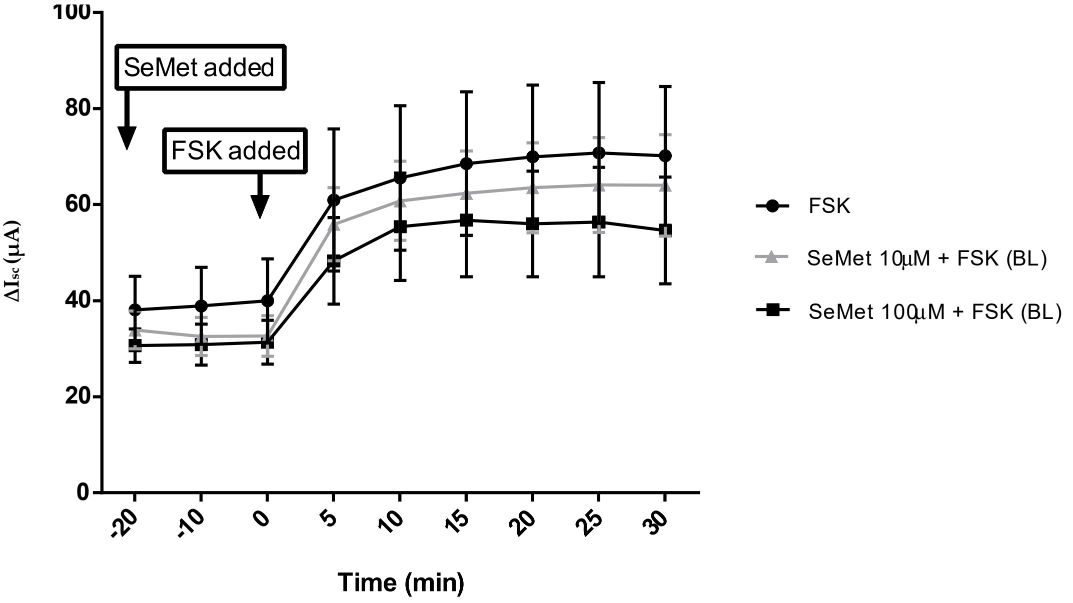


B

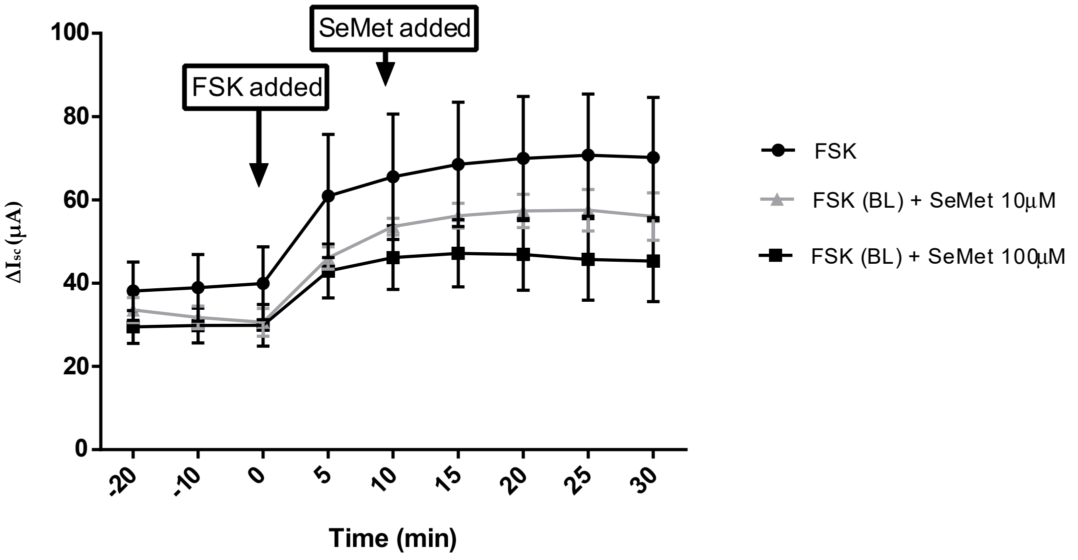


**Figure S1.** The effect of SeMet at 10μM and 100μM (AP&BL) on FSK-mediated $Cl^-$ secretion in rat jejunum mucosae. A) SeMet pre-incubated before FSK addition in jejunal mucosae; B) SeMet addition on plateau of FSK induced peak in jejunal mucosae.

**A**

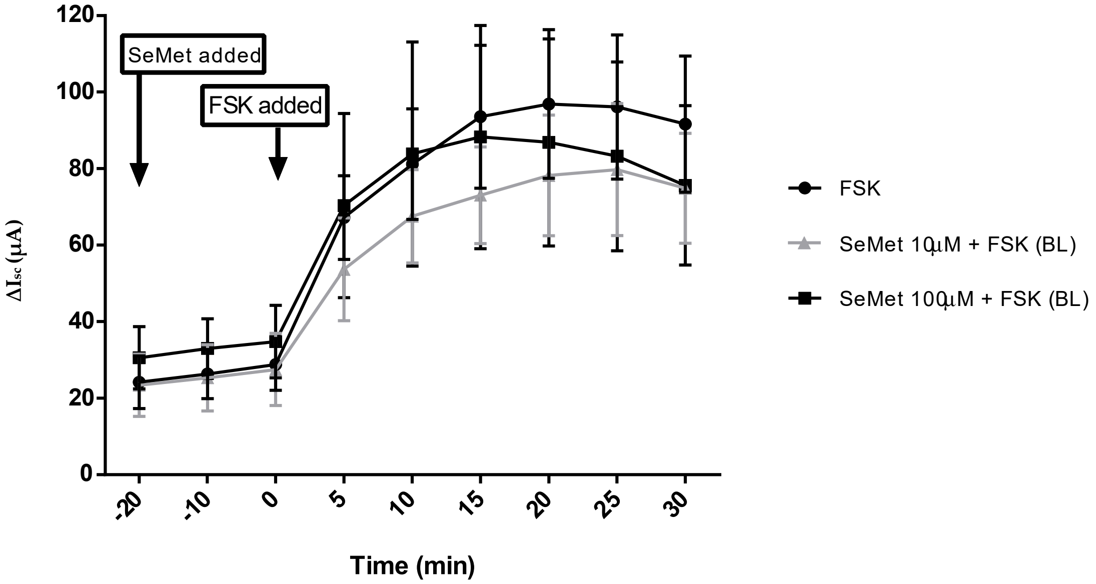


**B**

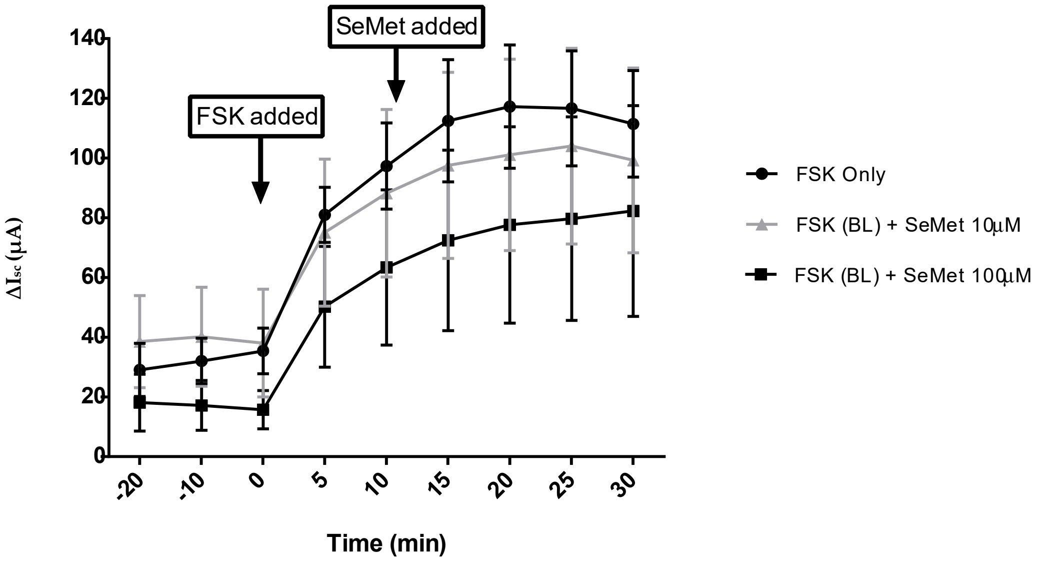


**Figure S2.** The effect of SeMet at 10μM and 100μM (AP&BL) on FSK-mediated $Cl^-$ secretion in rat colonic mucosae with A) SeMet pre-incubated before FSK addition in colonic mucosae; B) SeMet addition on plateau of FSK induced peak in colonic mucosae (n=6). 2-Way ANOVA with Bonferroni's post-test, no significance between treatments.

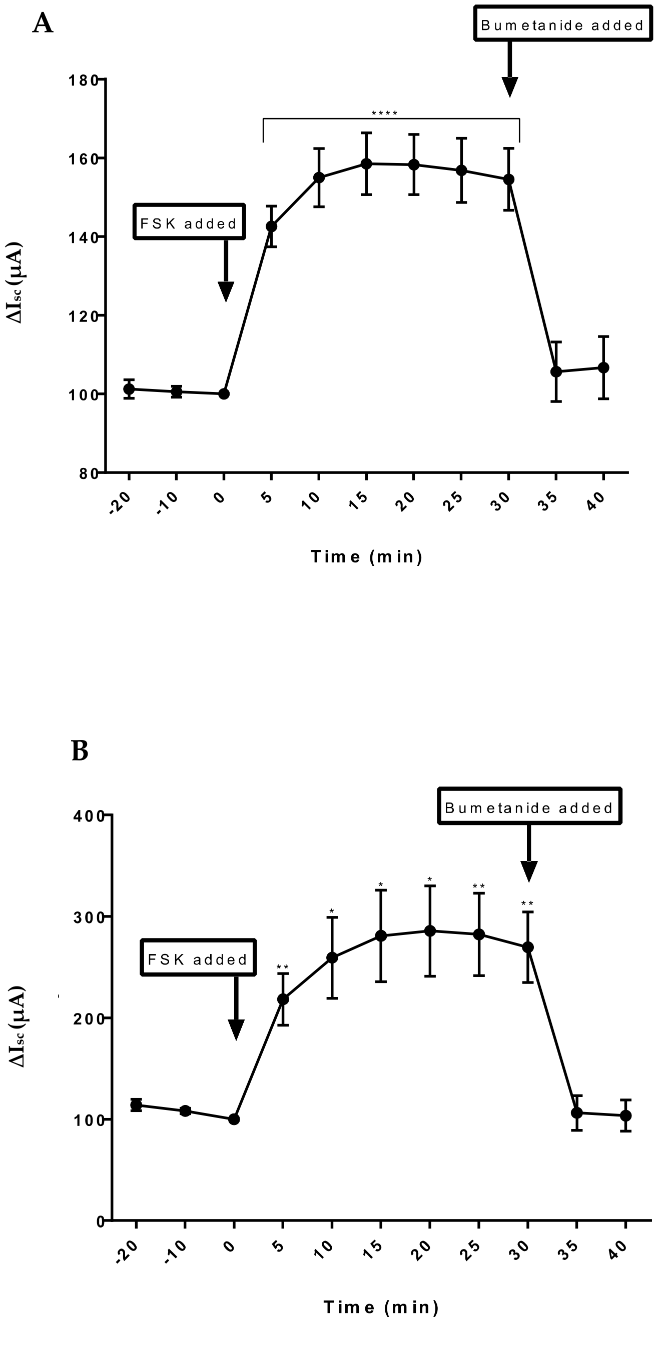


**Figure S3.** Bumetanide response to FSK stimulated $Cl^-$ secretion in A) jejunal and B) colonic mucosae measured by change in $I_{sc}$ compared to T=0 min. One-way ANOVA with Bonferroni's post-test multiple comparison; * P < 0.05, ** P < 0.01, and ***P< 0.001, ****P<0.0001 (N=16).
